# Exploratory Cluster Analysis to Identify Patterns of Chronic Kidney Disease in the 500 Cities Project

**DOI:** 10.5888/pcd15.170372

**Published:** 2018-05-17

**Authors:** Shelley H. Liu, Yan Li, Bian Liu

**Affiliations:** 1 Department of Population Health Science and Policy, Icahn School of Medicine at Mount Sinai, New York, New York; 2 Center for Health Innovation, New York Academy of Medicine, New York, New York

## Abstract

Chronic kidney disease is a leading cause of death in the United States. We used cluster analysis to explore patterns of chronic kidney disease in 500 of the largest US cities. After adjusting for socio-demographic characteristics, we found that unhealthy behaviors, prevention measures, and health outcomes related to chronic kidney disease differ between cities in Utah and those in the rest of the United States. Cluster analysis can be useful for identifying geographic regions that may have important policy implications for preventing chronic kidney disease.

## Objective

Chronic kidney disease (CKD) affects 15% of US adults (1). Although diabetes and high blood pressure are leading causes of CKD (1,2), other unhealthy behaviors and prevention behaviors also may play a role. The objective of this study was to determine how unhealthy behaviors, prevention measures, and outcomes related to CKD differ across US cities. We analyzed the cities at the state level, as literature shows that CKD prevalence and disease awareness differ across states (3). Understanding the state level differences may provide insight on policies and interventions to reduce CKD in urban settings.

## Methods

The Centers for Disease Control and Prevention (CDC) 500 Cities Project provides city-level data for 500 of the largest US cities for year 2014 (4,5). We merged socio-demographic data from the 2011–2015 American Community Survey 5-Year Estimates (6) with the CDC 500 Cities data by using matched census tracts. We created state-level prevalence estimates by averaging census-level socio-demographic data and city-level crude health measures from the 500 Cities Project, adjusting for population weight.

In our analysis, we included the following prevalence measures related to CKD: 5 unhealthy behaviors (binge drinking, current smoking, no leisure-time physical activity [LPA], obesity, and sleeping less than 7 hours); 3 prevention measures (no current health insurance, routine checkup within the last year, and taking blood pressure medication); and 3 outcome measures (CKD, diabetes, and high blood pressure).

To adjust for socio-demographic characteristics, we regressed each prevalence measure on the following confounding variables simultaneously using a beta regression (7): age (18–34 years, 35–64 years, ≥65 years), sex, race (white, Asian, black, other), ethnicity (Hispanic or Latino, not Hispanic or Latino), poverty (above/below poverty level), and education level (less than/more than high school). We used the regression residuals for each measure (hereafter “adjusted prevalence measures”) in the subsequent cluster analysis, as they indicate the portion of variability that cannot be explained by the socio-demographic characteristics. The adjusted prevalence measures were centered and scaled to make comparisons.

To identify differences between states, we implemented hierarchical cluster analysis (8–10) using the *hclust* function in R version 3.2.5 (free statistical computing software) with Euclidean distance as the distance measure and included the adjusted unhealthy behaviors and the prevention and outcome prevalence measures. Hierarchical clustering, which groups states on the basis of their mutual similarities, is a top-down approach in which all states are initially included in the same cluster, and the algorithm splits the states based on differences down the hierarchy. The algorithm ends when each state is in its own cluster.

## Results

**Figure 1:**
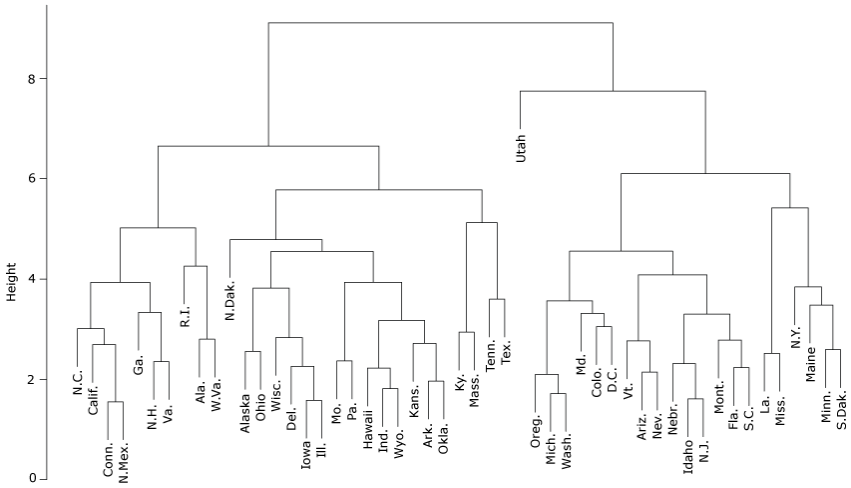
Cluster analysis of 500 US cities, summarized at the state level, plus Washington, DC, based on kidney disease–related factors (unhealthy behaviors, prevention measures, and outcomes related to CKD) and adjusted for socio-demographic characteristics.

Figure 1 shows the hierarchical relationship and partitions among the 500 cities, analyzed at the state level with the inclusion of Washington, DC. Clustering is depicted using a tree structure. The greater the height of the split, the more different are that state’s characteristics compared with other states. Meanwhile, states with splits at low heights are more similar to each other. The states initially split into two clusters of approximately equal size. Then Utah split from the other states in its cluster; the split height is high, meaning that Utah’s characteristics are dissimilar to those of other states. Meanwhile, several states have low split heights, indicating high similarity. These states are also geographically close to one another and share state borders: they include Arkansas and Oklahoma; Colorado and New Mexico; Arizona and Nevada; Iowa and Illinois; and Minnesota and South Dakota. We conducted a sensitivity analysis by varying the distance measure in our cluster analysis and found that the results are robust to different distance measures.

**Figure 2:**
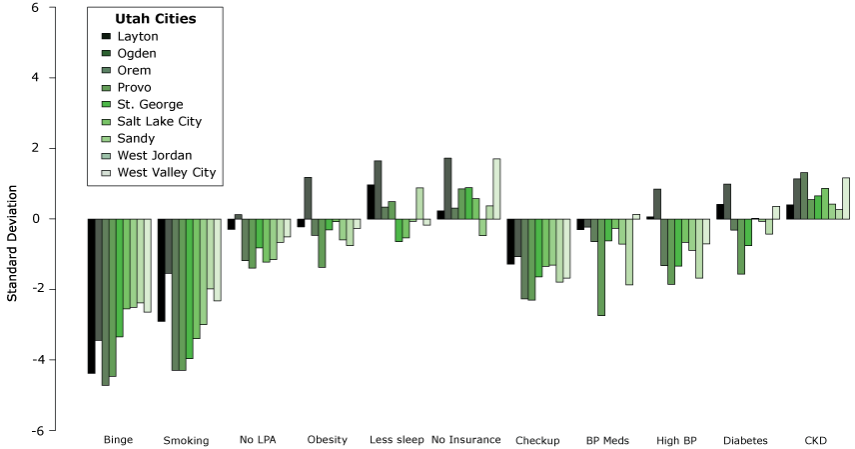
Variability of kidney disease–related factors^a^ in Utah cities compared with other cities in the United States. **Table:** 

Figure 2 shows how adjusted prevalence of unhealthy behaviors, prevention measures, and outcomes related to CKD differ between Utah cities and other US cities. We plotted the variability of these characteristics for the 9 Utah cities. Variability was quantified by the standard deviation (SD), comparing each city to other US cities in the 500 Cities Project. Adjusted prevalence of binge drinking and smoking were on average 3.4 and 3.1 SD lower, respectively, in Utah cities than in other US cities. Utah cities also had lower adjusted prevalence of annual physician checkup (1.6 SD) compared with the rest of the United States. Although adjusted prevalence of high blood pressure and diabetes were generally lower in Utah cities compared with the rest of the United States (0.84 and 0.15 SD, respectively), adjusted prevalence of chronic kidney disease was higher (0.76 SD) in all Utah cities.

Utah City	Binge	Smoking	No Leisure-Time Physical Activity	Obesity	Less Sleep	No Insurance	Check-up	Blood Pressure Meds	High Blood Pressure	Diabetes	Chronic Kidney Disease
**Layton**	**−4.41**	**−2.93**	**−0.30**	**−0.22**	**0.98**	**0.23**	**−1.29**	**−0.30**	**0.06**	**0.42**	**0.40**
**Ogden**	**−3.47**	**−1.56**	**0.12**	**1.19**	**1.66**	**1.74**	**−1.07**	**−0.23**	**0.86**	**1.00**	**1.15**
**Orem**	**−4.75**	**−4.32**	**−1.19**	**−0.47**	**0.34**	**0.31**	**−2.28**	**−0.65**	**−1.33**	**−0.31**	**1.33**
**Provo**	**−4.50**	**−4.33**	**−1.40**	**−1.38**	**0.50**	**0.86**	**−2.32**	**−2.76**	**−1.86**	**−1.57**	**0.55**
**St. George**	**−3.36**	**−3.99**	**−0.83**	**−0.31**	**−0.64**	**0.90**	**−1.65**	**−0.62**	**−1.35**	**−0.76**	**0.66**
**Salt Lake City**	**−2.56**	**−3.42**	**−1.23**	**−0.07**	**−0.54**	**0.58**	**−1.36**	**−0.27**	**−0.67**	**0.02**	**0.87**
**Sandy**	**−2.52**	**−3.01**	**−1.16**	**−0.60**	**−0.07**	**−0.48**	**−1.32**	**−0.72**	**−0.89**	**−0.07**	**0.43**
**West Jordan**	**−2.40**	**−2.00**	**−0.66**	**−0.76**	**0.89**	**0.38**	**−1.80**	**−1.89**	**−1.69**	**−0.43**	**0.27**
**West Valley City**	**−2.66**	**−2.34**	**−0.51**	**−0.27**	**−0.18**	**1.72**	**−1.69**	**0.14**	**−0.71**	**0.36**	**1.18**

Abbreviations: BMI, body mass index; BP, blood pressure; CKD, chronic kidney disease; LPA, leisure-time physical activity.

^a ^Factor definitions: Binge = binge drinking (reporting 5 or more drinks for men, or 4 or more drinks for women at an occasion in the last 30 days); Smoking = current smoking (have smoked over 100 cigarettes in their lifetime and currently smoke); No LPA = no leisure-time physical activity in the past month; Obesity = BMI ≥30; Less sleep = sleeping <7 hours; No insurance = no current health insurance coverage; Checkup = routine checkup within the past year; BP Meds = taking blood pressure medication; High BP = high blood pressure; Diabetes = has diabetes; CKD = has chronic kidney disease.

## Discussion

We identified clusters of 50 US states plus Washington, DC, based on the unhealthy behaviors, prevention measures, and CKD-related outcomes among adults living in cities. Among all states, Utah stood out because, compared with the national average among cities, cities in Utah had a higher adjusted prevalence of CKD but a lower adjusted prevalence of unhealthy behaviors. This information may be of interest to public health practitioners and policy makers in Utah, and suggests the need for further research to better understand which factors — beyond socio-demographic characteristics and well-known risk factors — could contribute to the prevalence of CKD. The cluster analysis also showed that several states in the South and Midwest that share geographical borders are clustered together, indicating cities in those states share similar characteristics.

This study had several limitations. First, because cluster analysis is a type of exploratory analysis, we could not provide causal inference or prediction based on the results. Second, because data from the 500 Cities Project originate from the Behavioral Risk Factor Surveillance System (BRFSS), which is self-reported, there may be social desirability bias, in which respondents may answer the survey in a way they believe may be viewed positively by others.

To our knowledge, this is one of the first articles to analyze data from the 500 Cities Project.
